# Endovascular model of ischemic stroke in swine guided by real-time MRI

**DOI:** 10.1038/s41598-020-74411-3

**Published:** 2020-10-14

**Authors:** D. Golubczyk, L. Kalkowski, J. Kwiatkowska, M. Zawadzki, P. Holak, J. Glodek, K. Milewska, A. Pomianowski, M. Janowski, Z. Adamiak, P. Walczak, I. Malysz-Cymborska

**Affiliations:** 1 Department of Neurosurgery, School of Medicine, Collegium Medicum, University of Warmia and Mazury, Warszawska 30, 10-082 Olsztyn, Poland; 2 Department of Radiology, Centre of Postgraduate Medical Education, Central Clinical Hospital of Ministry of the Interior and Administration in Warsaw, Warsaw, Poland; 3 Department of Surgery and Roentgenology With the Clinic, Faculty of Veterinary Medicine, University of Warmia and Mazury, Olsztyn, Poland; 4 Department of Internal Diseases With Clinic, Faculty of Veterinary Medicine, University of Warmia and Mazury, Olsztyn, Poland; 5 Center for Advanced Imaging Research and Department of Diagnostic Radiology and Nuclear Medicine, University of Maryland School of Medicine, Baltimore, MD USA

**Keywords:** Diseases of the nervous system, Neurological disorders, Experimental models of disease, Preclinical research

## Abstract

Modeling stroke in animals is essential for testing efficacy of new treatments; however, previous neuroprotective therapies, based on systemic delivery in rodents failed, exposing the need for model with improved clinical relevance. The purpose of this study was to develop endovascular approach for inducing ischemia in swine. To achieve that goal, we used intra-arterial administration of thrombin mixed with gadolinium and visualized the occlusion with real-time MRI. Placement of the microcatheter proximally to rete allowed trans-catheter perfusion of the ipsilateral hemisphere as visualized by contrast-enhanced perfusion MR scans. Dynamic T2*w MRI facilitated visualization of thrombin + Gd solution transiting through cerebral vasculature and persistent hyperintensities indicated occlusion. Area of trans-catheter perfusion dynamically quantified on representative slice before and after thrombin administration (22.20 ± 6.31 cm^2^ vs. 13.28 ± 4.71 cm^2^ respectively) indicated significantly reduced perfusion. ADC mapping showed evidence of ischemia as early as 27 min and follow-up T2w scans confirmed ischemic lesion (3.14 ± 1.41 cm^2^). Animals developed contralateral neurological deficits but were ambulatory. Our study has overcome long lasting challenge of inducing endovascular stroke model in pig. We were able to induce stroke using minimally invasive endovascular approach and observe in real-time formation of the thrombus, blockage of cerebral perfusion and eventually stroke lesion.

## Introduction

Ischemic stroke is a third leading cause of death and the leading cause of long-term serious disability in adults^1^. To date there is only one FDA-approved drug with demonstrated efficacy for ischemic stroke–alteplase (tPA), but with narrow therapeutic window (< 4.5 h), which makes majority of patients ineligible for this treatment^2^. Mechanical thrombectomy substantially extended the therapeutic window up to 24 h in selected patients, which increased the number of eligible patients^3^. Despite these positive developments burden of stroke remains high. Notably though, large cohorts of patients are now treated with placement of endovascular catheter in brain arteries thus there is renewed interest in complementing thrombectomy with adjuvant treatments targeting neuroinflammation or acting neuroprotectively to maximize benefits. Neuroprotective systemic therapies for stroke have been applied extensively in late nineties and while excellent efficacy has been shown in rodents every clinical trial failed^4–7^, exposing the problem of low clinical relevance of animal models.

Modeling stroke in animals provides important platform for studying disease and developing therapy, and, number of stroke animal models have been developed. More than 1000 therapeutics were tested in rodent models of ischemia^8,9^; however, differences between humans and rodents in brain structure are vast including lissencephalic vs. gyrencephalic organization, the size disparity up to 1000-fold or white matter content at 10% vs. 45% in humans^10^. Direct translation of results to humans, which is frequently practiced, does not seem appropriate in this case^11^. STAIR (Stroke Treatment Academic Industry Roundtable^12^) and STEPS ( Stem Cell Therapies as an Emerging Paradigm in Stroke^13^) committees suggest that clinical translation should be preceded by studies in large animal model of stroke.

Several approaches have been developed to induce stroke model in animals, and, as established in rodents, endovascular transient occlusion provides a model with a close resemblance to clinical stroke. Due to similarities in cerebral vasculature dogs are excellent for modeling stroke using endovascular approach^14^, but being companion animals are socially controversial as research subjects, and, in many countries experimentation using dogs is not allowed any more. Furthermore, mortality in dogs after endovascular stroke has been very high and likely due to herniation caused by prominent tentorium cerebelli and falx cerebri. The utility of primates is even more controversial. Domestic pig is considered excellent for modeling neurological disease because it has relatively large gyrencephalic brain, only seven times smaller than human brain and white–gray matter ratio and immune system closely resembles that in human^15^. Due to these positive features pig has been used for modeling of stroke; however, the only reliable stroke model in swine so far was based on surgical access to the middle cerebral artery (MCA). That approach is highly invasive with complex surgery in part due to the skull thickness including craniotomy and clipping or coagulating cerebral vessels^16^. Craniotomy is difficult and time consuming, severe morbidity of the procedure is highly undesired and reperfusion is not possible due to frequent hemorrhagic complications^17^. Previous attempts to induce stroke in swine, using endovascular approach was thwarted by complexity of cerebral vasculature with *rete mirabile*, a network of microvessels separating extracranial and intracranial circulation precluding advancing intra-arterial catheter into cerebral vessels for local obstruction of the cerebral blood flow^18^. Thrombus placement in the pharyngeal ascending artery, which is proximal to *rete* has been attempted but that did not produce cerebral ischemia^19^.

**Figure 1:**
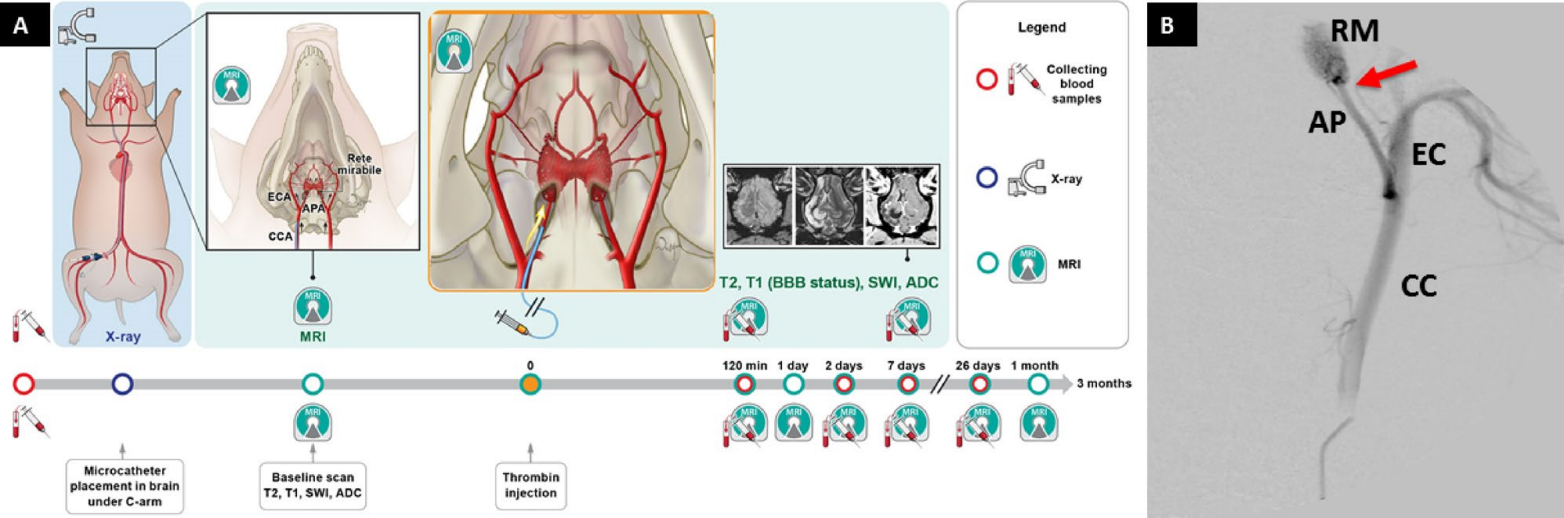
*Experimental Outline*. Timeline of experiments (**A**), Digital subtraction angiography with a catheter in APA (**B**; CC indicates common carotid artery, *EC* external carotid artery, *AP* ascending pharyngeal artery, *RM* rete mirabile, red arrow indicates placement of the microcatheter tip).

To overcome the limitation of *rete* mirabile and to develop endovascular model of stroke in swine we explored utility of interventional MRI and our previously developed strategies for visualization of a local trans-catheter cerebral perfusion to bypass the *rete* and induce occlusion in cerebral arteries. Advantage of ischemia induction inside MRI scanner was ability to dynamically monitor both the blockage of cerebral arteries and development of ischemia. Experimental approach is shown in the Fig. 1.

Overall aim of this study, that we successfully achieved, was to develop a model of stroke with high clinical relevance, using large animal, at low cost and at minimal ethical controversy. This model may be used for late stage pre-clinical testing of novel therapies.

## Results

### General observations

Stroke induction procedure, which lasted about 3–4 h was initially well tolerated by all nine animals. Three animals died during acute phase of stroke; one of them during induction of anesthesia for the follow-up scan, and, two of them were found dead 1 day after stroke and autopsy indicated extensive brain damage. Four animals were sacrificed 6 days post stroke induction, two additional were sacrificed three months later. All surviving animals were ambulatory, able to drink and eat independently though with marked neurological motor deficits in contralateral limbs. After stroke induction we observed rapid fatigue, animals were apathetic; usually tilting head to one side, had difficulties in walking, were confused, often exhibiting loss of balance and coordination.

### Intraarterial catheter placement

**Figure 2:**
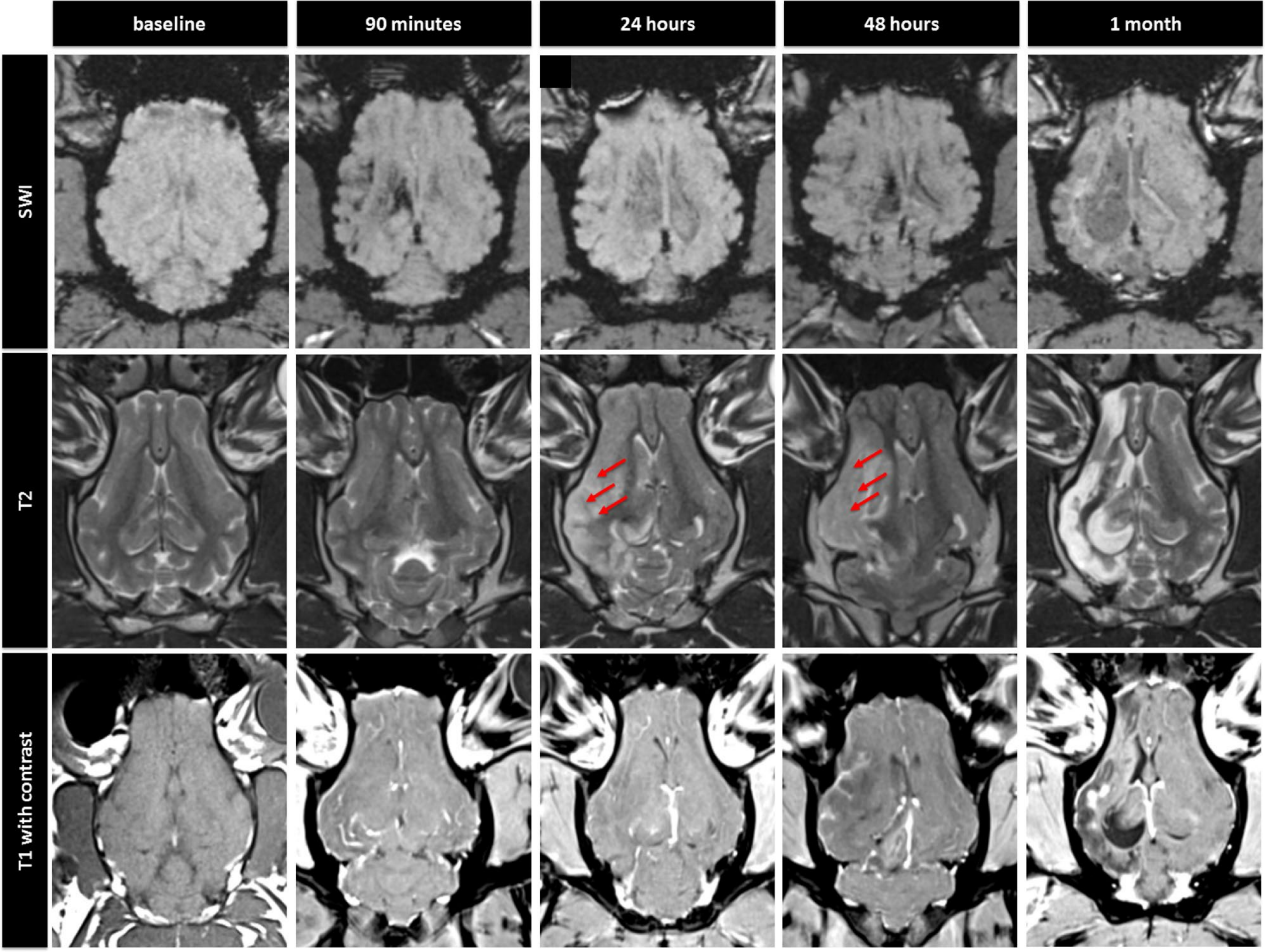
*Longitudinal MR imaging of representative animal showing evolution of acute* *stroke.* SWI, T2w (arrows indicate ischemic region) and T1 + Gd images at different time points.

After ultrasound-guided puncture and 5/6F introducer placement in femoral artery, non-braided 5F guiding catheter was advanced to common carotid artery under X-ray guidance. Using road map mode the catheter was navigated via common carotid artery to ascending pharyngeal artery with the tip placed just before the *rete mirabile* vessels. Digital subtraction angiography (DSA) confirmed proper placement of the catheter (Fig. 1B). Animals were transferred to 3 T MRI scanner and baseline scans did not show any focal abnormalities (Fig. 2) indicating that the catheter placement was safe.

**Figure 3:**
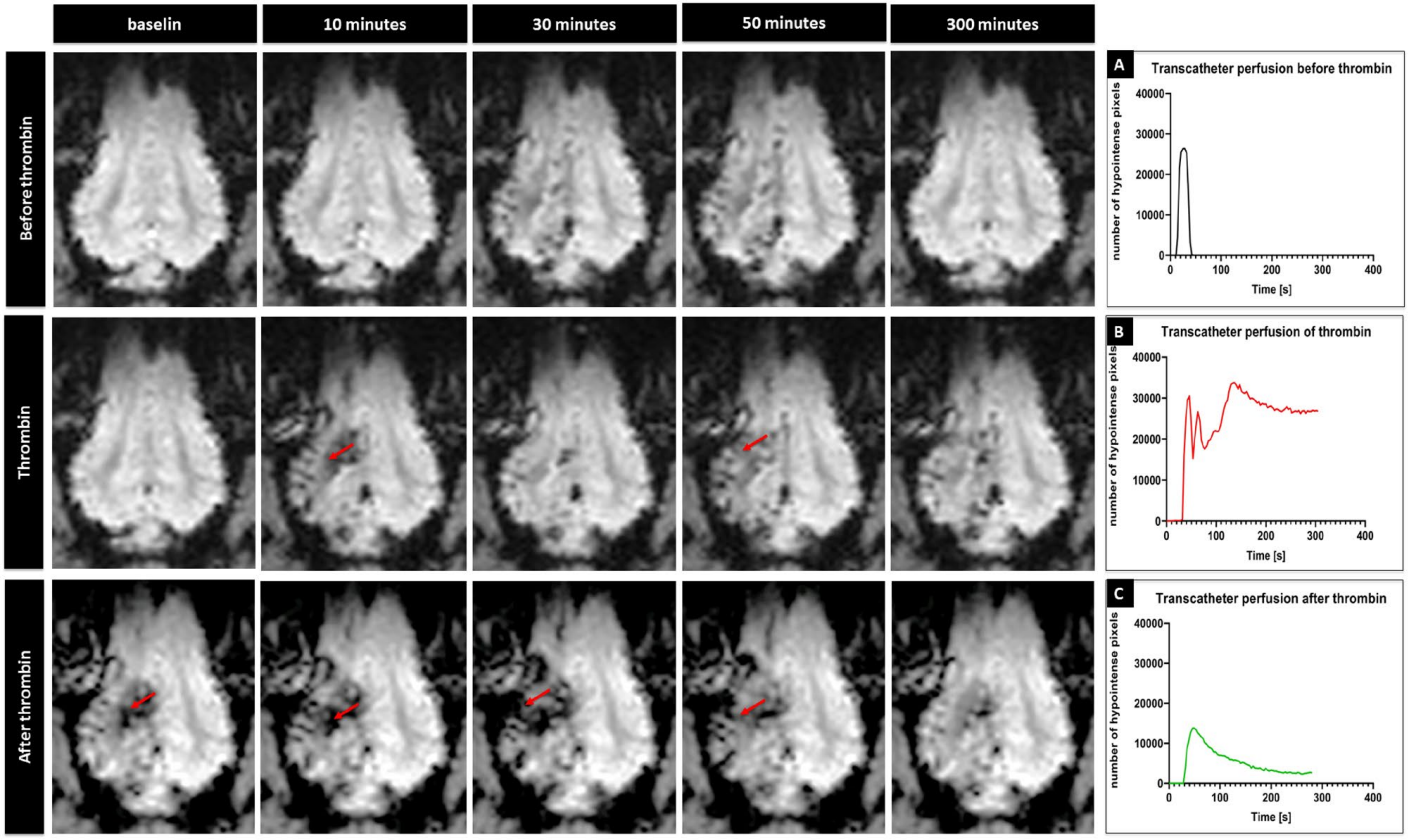
*Cerebral trans-catheter perfusion changes during induction of ischemia in representative animal.* Perfusion changes over time at baseline, thrombin mixed with contrast agent injection and thrombin injection of representative animal (red arrows point to hypointense regions) with quantification (**A**–**C**, respectively).

Contrast agent injection through the microcatheter with its tip placed in the APA was performed manually, under real-time MRI and imaging feedback made it possible to adjust infusion speed to optimize perfused brain area maximizing exposure of MCA territory (Fig. 3).

### Induction of ischemia under real-time MRI

**Figure 4:**
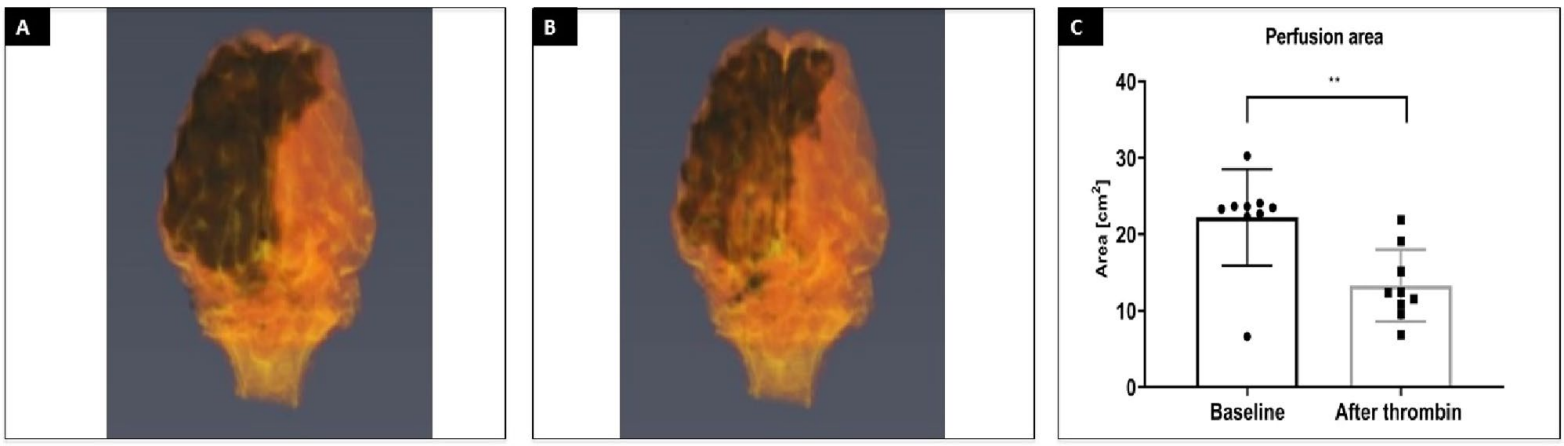
*3D rendering of cerebral trans-catheter perfusion territory.* 3D reconstruction of trans-catheter perfusion territory over time before (**A**) and after thrombin injection of representative animal (**B**) with quantification (**C**).

Intra-arterial injection of the contrast via microcatheter was used to assess cerebral perfusion in the brain before and after stroke induction as presence of catheter in APA precluded perfusion imaging with standard intravenous contrast agent delivery. Baseline contrast-enhanced perfusion scans showed brain territory supplied by the microcatheter infusion. 3D reconstruction of the perfused area showed rapid clearance of the contrast in all animals. Thrombin injection visualized in real-time on dynamic GE-EPI scans resulted in only minor vascular blockage after first bolus but second pulsating infusion was more effective with extensive retention of hyperintensity dynamically quantified in Fig. 3B. Perfusion area was visibly smaller after thrombin as shown with another contrast-enhanced perfusion scan (Fig. 3C). 3D reconstruction of the perfused area before and after thrombin injection is showed in Fig. 4A,B, respectively. Hypointense area in the ipsilateral hemisphere, measured on representative slice at the center of perfused area, decreased from 22.20 ± 6.31 cm^2^ at baseline to 13.28 ± 4.71 cm^2^ after thrombin injection (Fig. 4C). Number of hypointense pixels decreased by 40.2%. To measure blocking effect of thrombin on cerebral vasculature we collected dynamic MRI datasets during IA injection of Gadolinium before and after thrombin injection. Before thrombin (intact circulation) the contrast enters cerebral circulation (detected as hypointensity) and is rapidly cleared (within 17 s). After thrombin it took about 210 s to clear the contrast from the brain which is an indicator of severe blockage. In all animals we observed increase of time to clearance after thrombin injection.

### Early evolution of stroke monitored with ADC imaging

**Figure 5:**
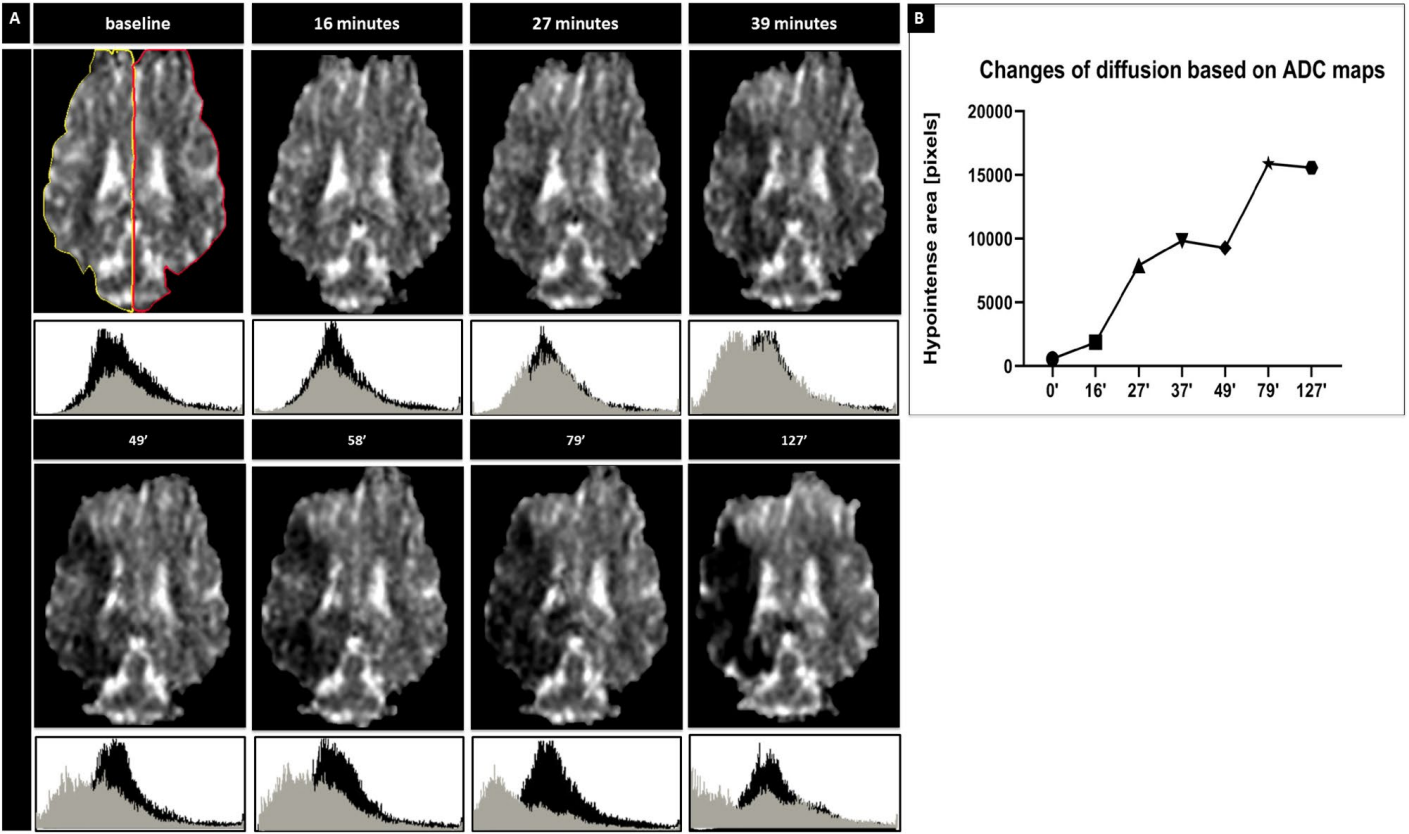
*Diffusion changes after stroke induction.* ADC over time with histograms for ipsilateral (grey) and contralateral (black) hemisphere (**A**). ROI for evaluation of changes in diffusion (**A**; yellow—ipsilateral and red—contralateral; X axis represents pixel intensity and Y axis represents number of pixels in that particular tone). ADC maps with quantification of brain area with abnormal ADC over time (**B**; apostrophes indicate minutes).

Changes of diffusion and particularly ADC maps are the most sensitive for early detection of ischemic damage. For quantitative assessment of stroke evolution, we generated signal intensity histograms for the ROIs encompassing the entire ipsilateral vs. contralateral hemisphere (Fig. 5) as well as for the entire brain for representative animal. First evidence of ischemic damage in brain parenchyma on diffusion was detected 27 min after intra-arterial thrombin injection with acceleration of damage observed between 16 and 27 min post clot induction (increase in ADC value in hypointense area) and continued expansion of stroke region between 49 and 79 min. After 2 h from induction of stroke we didn’t observe additional changes in diffusion on ADC maps.

### Longitudinal MRI assessment of the lesion

We found no abnormal T2 MRI signal for up to two hours after thrombin. Lesion consistent with ischemia was detected 24 h after thrombin injection on T2w scan as hyperintensity and edema (Fig. 2). Mean lesion size at the axial plain cutting through the center of the lesion shown in Fig. 2 was 3.14 ± 1.41 cm^2^ and lesion volume was 7.39 ± 5.41 cm^3^ (lesion size and volume of each animal is shown in the Supplementary Table S1). At this early stage of 24 h time point T1w images with contrast showed only marginal enhancement (Fig. 2) indicating no BBB breach or perhaps limited perfusion restricting access of the contrast agent. At the chronic stage, one month after induction of stroke, there was marked brain atrophy with T2 hyperintensity. T1 imaging with contrast showed distinct regions with persisting BBB opening.

### Histological evaluation of ischemic brain

**Figure 6:**
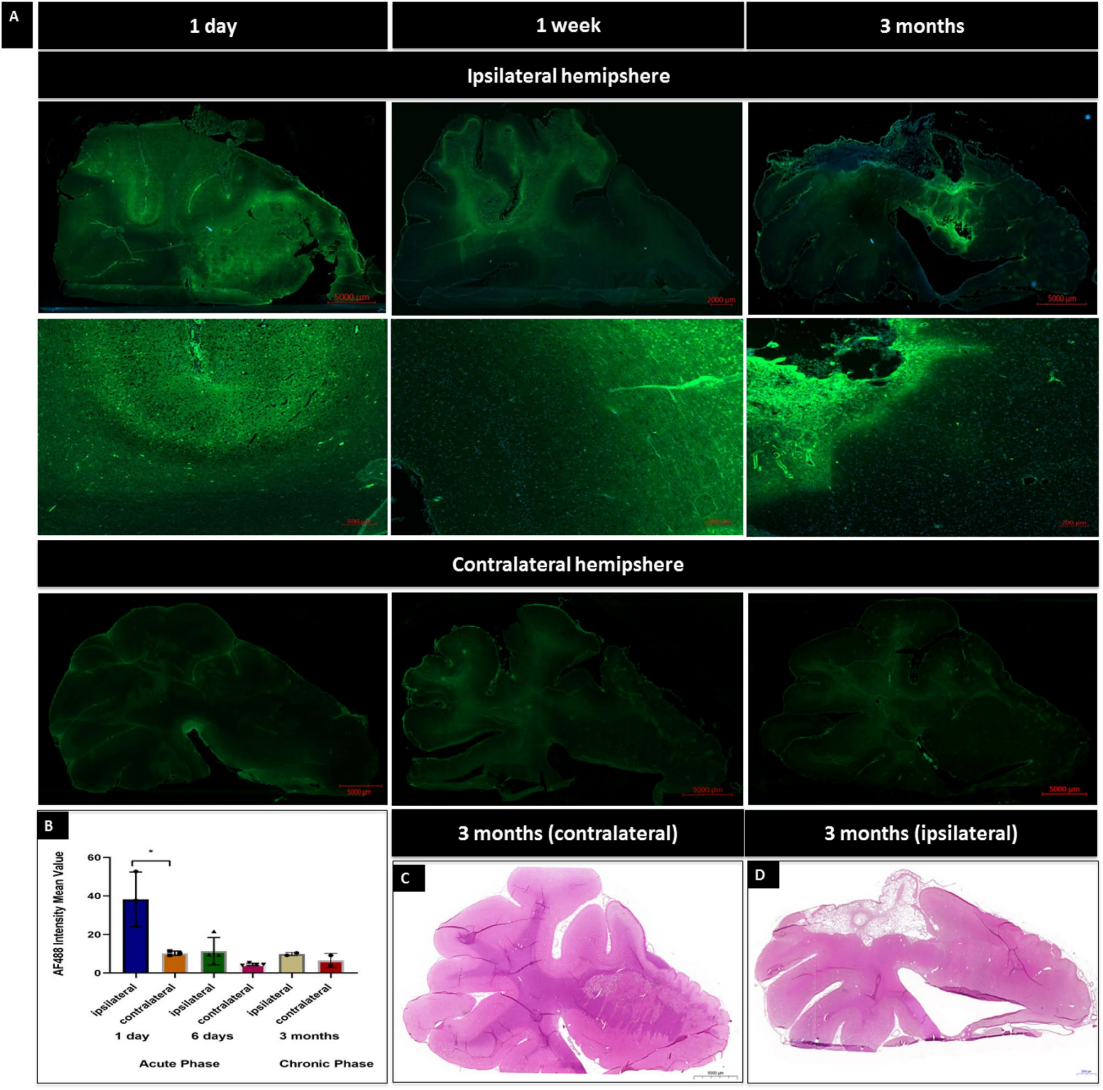
*Histological evaluation of stroke tissue.* BBB evaluation at 1 day, 1 week, and 3 months post stroke (**A**). Quantification of Intensity Mean Value (**B**). Hematoxylin and Eosin staining of the brain 3 months post stroke (**C**—contralateral hemisphere, **D**—ipsilateral hemisphere).

Histological hematoxylin & eosin staining (Fig. 6D) revealed a lesion corresponding in size and localization to that observed in MRI. Immunostaining for extravasating immunoglobulins with fluorescent anti-swine IgG showed elevated signal throughout the lesion indicating BBB breach. We observed BBB disruption during acute phase in pigs which died shortly after the procedure (Fig. 6A), after 6 days from the procedure (Fig. 6A) and even after 90 days post stroke (Fig. 6C). Quantification of anti-swine IgG AF488 intensity mean value showed higher level of fluorescence in the ipsilateral hemisphere in acute phase 1 day post stroke induction (38.23 ± 14.18) compared to the contralateral hemisphere (10.29 ± 1.117; Fig. 6B) with p value = 0.0273. Mean values of immunofluorescence in the chronic phase of stroke in contralateral hemisphere was at similar level (9.81 ± 0.85) compared to the contralateral hemisphere (6.58 ± 3.65; Fig. 6B) with p value = 0.3477.

To assess systemic effect of intraarterial thrombin injection we monitored thrombin time in blood samples. We observed increase in thrombin time from baseline (25.36 ± 7.623 s) to 41.80 ± 8.15 s after the procedure, however after day 5 thrombin time started to decrease (35.26 ± 8.17 s) and returned to baseline level after 26 days (Supplementary Fig. S1).

## Discussion

We have shown that occlusion of cerebral vasculature by intra-arterial injection of thrombin proximally to *rete mirabile* is feasible. It challenged a long-term paradigm that stroke cannot be induced in pigs using endovascular approach. Initially, there was high uncertainty whether blockage of perfusion occurs in *rete*, cerebral vessels or doesn’t occur at all. To address this issue we took advantage of our previously established interventional MRI approaches, which proved excellent for monitoring intra-arterial targeting^20^. Using that approach we were able to see in real-time during intervention if and when cerebral vessels have been blocked. Supplementation of thrombin with gadolinium-based contrast agent facilitated dynamic imaging and visualization of thrombus formation evidenced by persistence of hypointense signal after completed infusion. The location of thrombus has been then confirmed with high resolution SWI sequence. Thrombin/Gd solution infused at the constant speed cleared from the vasculature and pulsatile infusion induced clotting more effectively. MR imaging following thrombin injection demonstrated early changes in ADC, soon after the clot induction. Using that image-guided approach we were able to induce ischemia with high reproducibility. Our model has unique features and an advantage over existing craniotomy-based models of ischemic stroke. Bone removal and breach of the dura matter affects cerebral pressure, leads to loss of the cerebral-spinal fluid and increases the risk of infection. Animals after surgical stroke often require intensive care due to symptoms other than stroke-related neurological deficits^21^. Our method is relatively short, minimally invasive, precluding extensive stroke-independent morbidity due to surgical procedure and as such as improves clinical relevance. The time from skin incision to microcatheter placement in ascending pharyngeal artery was less than one hour. Time of the entire procedure was no longer than 4 h. Ischemic region was typically quite large and as expected this is related to relatively high mortality. This level of mortality can be related to edema and herniation evidenced by shift of the longitudinal cerebral fissure 24 h post stroke induction (shown in the Fig. 2). Mortality in craniectomy-based models is lower as trepanation mitigates effects of intracranial pressure peaks but as such it removes clinical relevance of surgical models. In our study two out of nine pigs developed severe morbidity after ischemia and died within 24 h. One additional animal was in relatively good condition at 24 h, but died during the follow-up MRI scan due to respiratory depression after anesthesia. This mortality at about 33% is comparable to that observed in surgical models or with rodent MCAO model^22–24^. This relatively high mortality rate could potentially be reduced by using lower concentration or reduced volume of intraarterially injected thrombin in future.

Sheep and pigs are mostly using craniotomy and craniectomy to induce stroke^16,25,26^. Few models of stroke exist using non-human primates, dogs, sheep or even pigs. Use of non-human primates are considered as controversial and in many countries impossible to perform because of restricted law. However, few models of ischemia exist in NHP^27,28^.

Endovascular model of stroke, besides rodents, has been reported for rabbits^29^, dogs^30^ and primates^31^—all characterized by favorable cerebral vasculature similar to humans. There were also attempts of performing endovascular procedures in swine^19^, but these were complicated by vascular *rete*. Jahan et al.^29^ developed selective middle cerebral artery (MCA) occlusion in rabbits using similar approach. They injected thrombin mixed with rabbit brain thromboplastin directly into the middle cerebral artery (MCA). MCA occlusion was achieved as confirmed with angiography. Jahan et al.^29^ did not evaluate whether the procedure results in ischemia, but they used this model to demonstrate pharmacological thrombolysis using plasmin delivered intraarterially. In dogs with much larger arteries there is more flexibility in navigating catheter and two routes for occluding canine MCA were reported with approach via internal carotid artery^32^ or via vertebral artery (VA)^33^. Stroke volume in dogs with ICA emboli injection varies depending on clot size and was the largest in group with clot 1.4 mm/1.7 mm combined with additional ipsilateral ICA transient occlusion (4173.23 ± 603.22 mm^3^)^14,32^. Despite the blood vessel collateralization within the *rete mirabile* in pigs or sheep there were few attempts to pass the embolic material beyond the *rete mirabile*. Ringer et al.^19^ showed that autologous thrombus was injected into ascending pharyngeal artery. They confirmed angiographically occlusion of APA; however, they didn’t perform any confirmation of ischemic injury which unlikely occurs considering contralateral contribution. Gralla^34^ used pigs as a model for mechanical thrombectomy in acute stroke. In this case they injected a clot into maxillary artery (MA) which led to far distal obstruction and tested possibilities of clot removal with clot retrieval device. Recent analysis by Herrmann et al.^35^ showed that only 2.7% of analyzed large animal studies used follow-up period up to 1 week and 36.5% used follow up between 3 and 6 months. Only 6.9% of studies were on large animals with the use of MRI to control for model performance. Our entire study was guided with imaging to assure precision, including navigation of the catheter under C-arm followed by MRI guidance to observe clot formation and stroke evolution over time. We followed up the animals for up to 3 months. Important advantages of our endovascular model of stroke compared to other reported large animal models include minimally invasive access, assessment of the very early stroke-related tissue changes with imaging, and potential for exploiting IA catheter placement to locally deliver adjuvant therapies, including stem cells^36^. The last feature is very attractive in the era of growing use of thrombectomy^37^. We observed quite high range of stroke lesion sizes with the mean infract volume 7.39 ± 5.41. However, this variability is consistent with clinically observed strokes as well as other swine studies^38^ showing mean lesion volumes at 18.25 ± 12.02 cm^3^.

We performed long term assessment of the BBB status and our IF data conclusively demonstrate BBB disruption at the chronic stage. This persisting BBB opening is likely associated with and contributes to the ongoing neuroinflammation, degeneration and astrocytic scar formation. This could be an important therapeutic target as some long-term patient follow-up studies report on BBB opening for up to 90 days after ischemic stroke^39^.

Rodents remain as the most widely used approach to study stroke^11^ and determine the efficacy of various drug therapies. However, inadequacy of these preclinical models has been considered as a major reason for unsuccessful translation of experimental stroke therapies into the clinic^12^. One example of that discrepancy is with cell transplantation approaches. Pre-clinical rodent experiments show frequently significant functional recovery and reduction in MCAO lesion volume such as following intravenous injection MSCs^40,41^. However, clinical trials following these protocols show marginal or lack of benefit^42,43^ The issue of animal model adequacy has been tackled by the Stroke Treatment Academic Industry Roundtable (STAIR) committee by releasing their recommendations. According to these recommendations, preclinical studies should be established with animal model that mimics clinical ischemic stroke, efficacy and safety of tested therapy should be verified in at least two species. Rodents due to ease in handling can be used for initial screening with strong recommendation for validation in large animal model^12^.

There are some limitations of this study. Firstly, we focused here on the feasibility of induction of cerebral ischemia without reperfusion. Further studies are needed to the extent of reperfusion and whether this model is suitable for induction of pharmacological thrombolysis using either intra-arterial or intravenous route. We do anticipate however that pharmacological thrombolysis will be effective in this model as freshly induced thrombi should be easier to dissolve compared to more organized thrombotic material dislodged from distant sites. Studies on reperfusion in this model will be important particularly considering growing role of mechanical endovascular thrombectomy and perspective for using intraarterial adjuvant treatments after reperfusion. On the other hand, meta-analysis by Cui et al.^44^ showed the need for clinically relevant models of permanent occlusion. Secondly, this study was performed in young animals as mature or aging pigs are difficult to manage in such study. Use of older minipigs could be a good solution. Access to advanced imaging equipment and neuroradiology expertise may be a limitation; however, with growing interest in endovascular treatment of stroke access to the infrastructure and expertise is improving. Thirdly, this study was performed on a relatively small group of animals. We were able to demonstrate with this number of animals how to overcome the challenge of rete mirabile for induction of stroke but obviously further testing is needed to confirm feasibility, reproducibility and utility of the model for studies on reperfusion and novel therapeutic approaches.

In summary, this study has provided for the first time feasibility of induction of endovascular stroke model in pig. We were able to observe in real-time formation of thrombin-induced clot and resulting blockade of cerebral perfusion and eventually stroke lesion. Our model produced relatively large infarct volume covering majority of MCA territory. Overall, we have established a new model of ischemic stroke in pigs with high clinical relevance. The model will contribute to studies aimed at understanding stroke pathophysiology and most of all development of new drugs.

## Materials and methods

The data that support the findings of this study are available from the corresponding author upon reasonable request.

### Experimental animals

Animal procedures were approved by local ethics committee of University of Warmia and Mazury in Olsztyn (54/2018 and 45/2019) and were performed according to ARRIVE guidelines. Nine juvenile (5 month old) male, domestic pigs (Polish Large White, average weight 40 kg) were used in this study. Animals were acclimated for at least two weeks at the housing facility before initiating the procedures. Animals had access to water and food ad libitum*.*

### Surgery

Surgeries were performed in a dedicated large animal surgical suite. Animals were divided into two groups according to the time of sacrifice (7 days and 3 months). Animals were pre-anesthetized with atropine (0.05 mg/kg i.m., Polfa, Poland), xylazine (3 mg/kg i.m., Vet-Agro, Poland) and ketamine (6 mg/kg i.m., Biowet—Puławy, Poland) and anesthetized by propofol (5 mg/kg/h i.v., B.Braun Melsungen AG, Germany) and sevoflurane (1–3%, Abbvie, Poland). Animals received butophanol every 4 h (0.2 mg/kg i.m., Zoetis, Poland). Femoral artery was identified using ultrasonography. Sheath Introducer (5F, Terumo) was placed transdermally in femoral artery. Using this port, endovascular catheter (Vertebral 5F, 110 cm, Balton), was placed and using hydrophilic guidewire (Merit Laureate, Merit Medical) was navigated to the arch of aorta and then to the common carotid artery. In the outlet of ascending pharyngeal artery (APA) microcatheter (UltraFlow HPC Flow Directed Micro Cathether, ev3) navigated over microguidewire (0.008″, Balt) was placed under C-arm guidance proximally to the *rete mirabile*. Continuous flushing with heparinized (5000 U/l) saline was maintained to avoid occlusion of the microcatheter. All vital parameters (saturation, heart rate, pressure, respiratory rate) were monitored during the entire procedure. Before and after procedure as well as during MRI follow-ups we collected blood samples for thrombin time (TT) analysis.

### Magnetic resonance imaging

Animals with microcatheter secured in the APA were transferred to 3 T MRI scanner (Magnetom Trio, Siemens). MRI protocol included dynamic GE-EPI (TE/TR = 30/1750) for assessment of trans-catheter cerebral perfusion, GE-EPI for monitoring thrombin-mediated blood clotting as well as SWI (TE/TR = 20/28), diffusion, T2w (TE/TR = 83/5660) and T1w with contrast (TE/TR = 3.69/20). Under real-time MRI guidance (GE-EPI scan) thrombin solution (Biomed, Poland) mixed with gadolinium contrast (Bayer, Germany) agent (1:20) was injected intra-arterially via microcatheter with a pulsatile mode over 1 min. Perfusion area (pixel intensity changes) were analyzed with Matlab software (MathWorks Inc., USA) quantifying number of hypointense pixels during infusion of the contrast or contrast/thrombin mixture. MRI follow up was performed 1, 3, 7 days and 1-month post stroke induction. Intraarterial trans-catheter perfusion scans, before and after thrombin injection, were analyzed using AMIRA 6.4 (Thermo Fisher Scientific—FEI) to do the 3D reconstruction of trans-catheter perfusion territory over time. We used arithmetic function to perform calculations on a pair of input data objects: 1. baseline, which was a dynamic susceptibility contrast-enhanced dataset acquired before intraarterial bolus of a contrast agent, and 2. dataset during intraarterial bolus of a contrast. According to a user-defined arithmetic expression (B-A) pixels with reduction in signal intensity were segmented and visualized using volren module. For calculations of a lesion volume and diameter Horos software was used. The boundary of the T2 hyperintense lesion area was manually outlined with pencil tool and diameter of the lesion on each slice as well as volume were measured.

### Sacrifice

After the last follow-up (7 days or 3 months) animals were pre-anesthetized as above and received lethal dose of sodium pentobarbital (Euthasol, Fatro, Poland). Animals were transcardially perfused with 5% sucrose followed by 4% PBS-buffered paraformaldehyde (PFA). Perfusion pressure was maintained at 120–140 mmHg. Brains were harvested and post-fixed in 4% PFA for 48 h. Brains were divided into 6 blocks (right, left and anterior, middle and posterior), cryo-protected in 30% sucrose until sank, frozen on dry ice powder for five minutes and kept in − 70 °C until further analysis. Brain of animals found dead after the procedure was extracted and cryo-protected as above.

### Histology

Samples were cryosectioned on a Hyrax C25 PLMC cryostat (Zeiss, Germany) as 10-μm coronal sections, mounted on microscopy slides, and stored at − 20 °C until histological evaluation.

#### H&E

For Hematoxylin & Eosin staining, sections were rehydrated in a graded series of EtOH (ethanol; 95%, followed by 80%, 70% EtOH and distilled water), for 3 min per each at room temperature. Then sections were rinsed in distilled water and transferred for 10 min to hematoxylin solution followed by 10 min in running water. Sections were differentiated in acidified alcohol and washed for 5 min in running tap water. Next, sections were transferred to eosin solution for three minutes, rinsed in distilled water and hydrated in 70%, 95%, and 100% ethanol, overexposed in xylene (2 times for 10 min) and mounted using DPX mounting medium (Sigma Aldrich). Images were captured using 3DHISTECH scanner.

#### Immunohistochemistry

For immunofluorescent staining, slides were left to dry at room temperature for 30 min and washed three times in PBS. Tissue sections were blocked with 10% NGS (Normal Goat Serum) in PBS. Anti-swine IgG Alexa Fluor 488 (1:100, Jackson ImmunoResearch Labotory, USA) was used to assess status of the Blood–Brain Barrier (BBB). After the blocking slide-mounted tissues were incubated with antibodies overnight in 4 °C. Next day antibodies were removed and sections were coversliped with antifade mounting medium containing DAPI (Fluoroshield, Sigma Aldrich, Germany). Images were captured using Zeiss Axio Observer microscope (Zeiss, Germany) and analyzed using ZEN 2.3 pro software (Zeiss, Germany). As our region of interest (ROI) we selected areas inside of the lesion (ipsilateral hemisphere and corresponding structure in the intact contralateral hemisphere. Tissue was outlined using a parametric active contour method (spline contour) and AF488 intensity mean values were measured.

### Statistical analysis

Shapiro–Wilk test was performed for check normality of the results. Statistical analysis of fluorescence mean intensity in acute phase of stroke (Mann–Whitney test) and perfusion area (unpaired t-test) was performed using GraphPad Prism 5.0 (GraphPad Software, Inc, San Diego, CA). All numerical data are presented as the mean ± standard deviation, and differences were considered as statistically significant at the 95% confidence level (p < 0.05).

## Supplementary information

Supplementary Information.

## Data Availability

The datasets generated and/or analysed during the current study are available from the corresponding author on reasonable request.

## References

[1] Benjamin EJ (2018). Heart disease and stroke statistics-2018 Update: A report from the American Heart Association. Circulation.

[2] National Institute of Neurological Disorders and Stroke rt-PA Stroke Study Group (1995). Tissue plasminogen activator for acute ischemic stroke. N. Engl. J. Med..

[3] Goyal M (2016). Endovascular thrombectomy after large-vessel ischaemic stroke: A meta-analysis of individual patient data from five randomised trials. Lancet.

[4] Yasuhara T (2009). Notch-induced rat and human bone marrow stromal cell grafts reduce ischemic cell loss and ameliorate behavioral deficits in chronic stroke animals. Stem Cells Dev..

[5] Steinberg GK (2018). Two-year safety and clinical outcomes in chronic ischemic stroke patients after implantation of modified bone marrow-derived mesenchymal stem cells (SB623): A phase 1/2a study. J. Neurosurg..

[6] Kidwell CS (2013). A trial of imaging selection and endovascular treatment for ischemic stroke. N. Engl. J. Med..

[7] Broderick JP (2013). Endovascular therapy after intravenous t-PA versus t-PA alone for stroke. N. Engl. J. Med..

[8] Jickling GC, Sharp FR (2015). Improving the translation of animal ischemic stroke studies to humans. Metab. Brain Dis..

[9] O'Collins VE (2006). 1,026 experimental treatments in acute stroke. Ann. Neurol..

[10] Boltze J (2017). Concise review: Increasing the validity of cerebrovascular disease models and experimental methods for translational stem cell research. Stem Cells.

[11] Cui LL, Golubczyk D, Jolkkonen J (2017). Top 3 behavioral tests in cell therapy studies after stroke: Difficult to stop a moving train. Stroke.

[12] Fisher M (2009). Update of the stroke therapy academic industry roundtable preclinical recommendations. Stroke.

[13] Savitz SI (2011). Stem cell therapy as an emerging paradigm for stroke (STEPS) II. Stroke.

[14] Atchaneeyasakul K (2016). Large animal canine endovascular ischemic stroke models: A review. Brain Res. Bull..

[15] Felix B (1999). Stereotaxic atlas of the pig brain. Brain Res. Bull.

[16] Platt SR (2014). Development and characterization of a Yucatan miniature biomedical pig permanent middle cerebral artery occlusion stroke model. Exp. Transl. Stroke Med..

[17] Al-Jehani H, Petrecca K, Martel P, Sinclair D, Sirhan D (2016). Decompressive craniectomy for ischemic stroke: Effect of hemorrhagic transformation on outcome. J. Stroke Cerebrovasc. Dis..

[18] Burbridge B, Matte G, Remedios A (2004). Complex intracranial arterial anatomy in swine is unsuitable for cerebral infarction projects. Can. Assoc. Radiol. J..

[19] Ringer AJ, Guterman LR, Hopkins LN (2004). Site-specific thromboembolism: A novel animal model for stroke. Am. J. Neuroradiol..

[20] Walczak P (2017). Real-time MRI for precise and predictable intra-arterial stem cell delivery to the central nervous system. J. Cereb. Blood Flow Metab..

[21] Boltze J (2008). Permanent middle cerebral artery occlusion in sheep: A novel large animal model of focal cerebral ischemia. J. Cereb. Blood Flow Metab..

[22] Fukuda Y (2015). Intra-arterial transplantation of low-dose stem cells provides functional recovery without adverse effects after stroke. Cell Mol. Neurobiol..

[23] Jiang C (2013). Comparison of the therapeutic effects of bone marrow mononuclear cells and microglia for permanent cerebral ischemia. Behav. Brain Res..

[24] Ishizaka S (2013). Intra-arterial cell transplantation provides timing-dependent cell distribution and functional recovery after stroke. Stroke.

[25] Mattingly TK (2016). Catheter based selective hypothermia reduces stroke volume during focal cerebral ischemia in swine. J. Neurointerv. Surg..

[26] Imai H (2006). A new model of focal cerebral ischemia in the miniature pig. J. Neurosurg..

[27] Chen X (2015). An ischemic stroke model of nonhuman primates for remote lesion studies: A behavioral and neuroimaging investigation. Restor. Neurol. Neurosci..

[28] Huang J (2000). A modified transorbital baboon model of reperfused stroke. Stroke.

[29] Jahan R (2008). Middle cerebral artery occlusion in the rabbit using selective angiography: Application for assessment of thrombolysis. Stroke.

[30] Chung DJ (2009). Intraarterially delivered human umbilical cord blood-derived mesenchymal stem cells in canine cerebral ischemia. J. Neurosci. Res..

[31] Kito G (2001). Experimental thromboembolic stroke in cynomolgus monkey. J. Neurosci. Methods.

[32] Zu QQ (2013). An endovascular canine stroke model: middle cerebral artery occlusion with autologous clots followed by ipsilateral internal carotid artery blockade. Lab. Investig..

[33] Zhang Y (2015). A novel canine model of acute vertebral artery occlusion. PLoS ONE.

[34] Gralla J (2006). A dedicated animal model for mechanical thrombectomy in acute stroke. Am. J. Neuroradiol..

[35] Herrmann AM (2019). Large animals in neurointerventional research: A systematic review on models, techniques and their application in endovascular procedures for stroke, aneurysms and vascular malformations. J. Cereb. Blood Flow Metab..

[36] Guzman R, Janowski M, Walczak P (2018). Intra-arterial delivery of cell therapies for stroke. Stroke.

[37] Evans MRB, White P, Cowley P, Werring DJ (2017). Revolution in acute ischaemic stroke care: A practical guide to mechanical thrombectomy. Pract. Neurol..

[38] Zhang R, Bertelsen LB, Flo C, Wang Y, Stodkilde-Jorgensen H (2016). Establishment and characterization of porcine focal cerebral ischemic model induced by endothelin-1. Neurosci. Lett..

[39] Naqvi I, Hitomi E, Leigh R (2019). Sustained opening of the blood-brain barrier with progressive accumulation of white matter hyperintensities following ischemic stroke. Brain Sci..

[40] Omori Y (2008). Optimization of a therapeutic protocol for intravenous injection of human mesenchymal stem cells after cerebral ischemia in adult rats. Brain Res..

[41] Komatsu K (2010). Therapeutic time window of mesenchymal stem cells derived from bone marrow after cerebral ischemia. Brain Res..

[42] Bang OY, Lee JS, Lee PH, Lee G (2005). Autologous mesenchymal stem cell transplantation in stroke patients. Ann. Neurol..

[43] Prasad K (2014). Intravenous autologous bone marrow mononuclear stem cell therapy for ischemic stroke: A multicentric, randomized trial. Stroke.

[44] Cui LL, Golubczyk D, Tolppanen AM, Boltze J, Jolkkonen J (2019). Cell therapy for ischemic stroke: Are differences in preclinical and clinical study design responsible for the translational loss of efficacy?. Ann. Neurol..

