# Supplementary material for: Endovascular model of ischemic stroke in swine guided by real-time MRI
Source: Sci Rep. 2020 Oct 14;10:17318. doi: 10.1038/s41598-020-74411-3 (PMC7560864; doi:10.1038/s41598-020-74411-3)
Supplement: Supplementary file 1 — Supplementary Information. [file 41598_2020_74411_MOESM1_ESM.docx]

**Endovascular model of ischemic stroke in swine guided by real-time MRI**

Golubczyk D., MSc ^1^ ; Kalkowski L., MSc ^1^ ; Kwiatkowska J., PhD^1^; Zawadzki M., MD/PhD^2^; Holak P., DVM, PhD^3^; Glodek J., DVM, PhD ^3^; Milewska K., MSc ^1^; Pomianowski A.,DVM.^4^; Janowski M., MD/PhD ^5^; Adamiak Z, DVM ^3^; Walczak P., MD/PhD ^5^, Malysz-Cymborska I., PhD ^1,*^

^1^ Department of Neurosurgery, School of Medicine, Collegium Medicum, University of Warmia and Mazury, Olsztyn, Poland

^2^ Department of Radiology, Centre of Postgraduate Medical Education, Central Clinical Hospital of Ministry of the Interior and Administration in Warsaw, Poland

^3^ Department of Surgery and Roentgenology with the Clinic, Faculty of Veterinary Medicine,

University of Warmia and Mazury, Olsztyn, Poland

^4^ Department of Internal Diseases with Clinic, Faculty of Veterinary Medicine, Faculty of Veterinary

Medicine, University of Warmia and Mazury, Olsztyn, Poland

^5^ Center for Advanced Imaging Research and Department of Diagnostic Radiology and Nuclear Medicine, University of Maryland School of Medicine, Baltimore, MD, USA

***To whom correspondence should be addressed:**

Department of Neurosurgery

School of Medicine Collegium Medicum

University of Warmia and Mazury in Olsztyn

Warszawska 30,

10-082 Olsztyn

Phone:+48 605 118 887

[i.m.cymborska@gmail.com](mailto:i.m.cymborska@gmail.com)

**Supplementary Table 1.** Lesion size and volume of each individual

| Pig no. | Latest measurement time point | Largest lesion diameter [cm^2^] | Lesion volume [cm^3^] |
| --- | --- | --- | --- |
| 1 | 3 months | 6,43 | 10,74 |
| 2 | 3 months | 3,67 | 2,24 |
| 3 | 7 days | 7,41 | 8,98 |
| 4 | 7 days | 3,53 | 2,87 |
| 5 | 7 days | 1,36 | 0,66 |
| 6 | 7 days | 7,90 | 14,49 |
| 7 | 1 day * | 6,55 | 11,77 |

* died during induction of anesthesia 24 hours after stroke


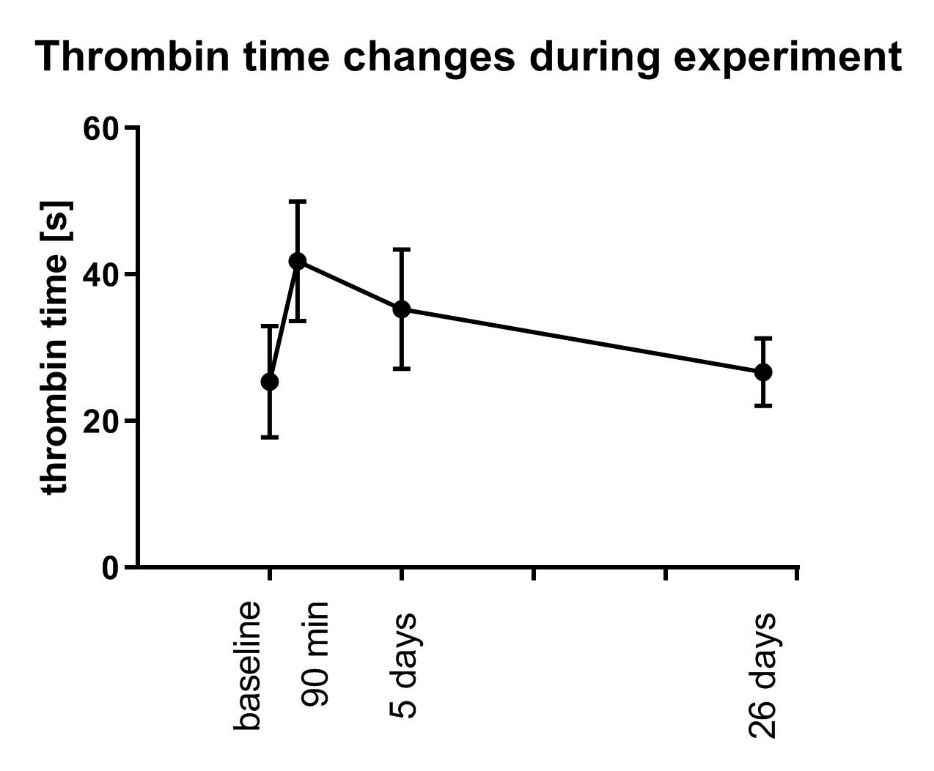


**Supplementary Figure 1**. Thrombin time evaluation over time.
